# In-vivo visualisation of the anatomical structures related to the acupuncture points Dai mai and Shen mai by MRI: A single-case pilot study

**DOI:** 10.1186/1471-2342-7-4

**Published:** 2007-03-14

**Authors:** Roy Moncayo, Ansgar Rudisch, Markus Diemling, Christian Kremser

**Affiliations:** 1 WOMED, Karl-Kapferer-Strasse 5, 6020 Innsbruck, Austria; 2 Innsbruck Medical University, Dept. of Radiology I, Innsbruck, Austria; 3 Hermes Medical Solutions AB, Skeppsbron 44, 111 30 Stockholm, Sweden

## Abstract

**Background:**

The concept of acupuncture point localisation in Traditional Chinese Medicine (TCM) is based on millenary practical experience. Modern imaging methods such as PET, MRI and SPECT have been used primary for the investigation of the mechanisms of action of acupuncture. In this pilot single-case study we have evaluated the technical possibilities for in-vivo imaging of the anatomical relations of acupuncture points using state of the art MRI.

**Methods:**

Preliminary experiments relating to the quality of acupuncture needles under the setting of MRI were done both with stainless steel and gold needles. In a second step, in-vivo imaging was carried out. A licensed acupuncture practitioner (RM) chose two points belonging to the so-called extraordinary vessels. In 2 sequential, separate procedures, he inserted himself gold acupuncture needles using a neutral technique (known as Ping Bu Ping Xie) into the Dai mai and Shen mai points, i.e. gall bladder 26 and bladder 62. Imaging was done on a Siemens Magnetom Avanto MR scanner using a head array and body coil. Mainly T1-weighted imaging sequences, as routinely used for patient exams, were used to obtain multi-slice images.

**Results:**

In the preliminary experiments only acupuncture needles made of gold showed enough stability in order to be used for further imaging procedures. Using an onion and a banana as an object, further studies showed that the gold needles produced a void defect that corresponds to the tip of the inserted needle, while at the same time an artefactually increased diameter was observed. The in-vivo experiments showed that the Dai mai point was in relation to the abdominal internal oblique muscle. The Shen mai point artefact showed up close to the longus and brevis peroneal tendons at the fibular malleolus. Side effects related to heating or burning were not observed. Improved anatomical recognition was obtained using 3D-volume rendering techniques.

**Conclusion:**

Through an adequate choice of acupuncture material (gold needles) as well as of ideal MRI imaging sequences it has been possible to visualize the anatomical characteristics at the acupuncture points Dai mai and Shen mai in-vivo. At the selected sites the needles showed a relation to tendino-fascial and muscular structures. These anatomical structures fit well into the recently described WOMED concept of lateral tension in which these acupuncture points play a regulatory role.

## Background

The neurophysiological model of acupuncture mechanisms has been matter of research in the past years. In this quest, imaging methods such as PET, SPECT and fMRI have been used to investigate changes of brain perfusion or activation in response to acupuncture techniques [1-3]. In spite of these advances little has been done for the in-vivo characterization of the acupuncture points using modern imaging techniques.

Attempts to characterize acupuncture points have been made by radionuclide imaging. Lazorthes et al. investigated several acupuncture points (LI4, LI11, LI14, SI3, TH5, GB34, Ren mai 12, and Du mai 4). The most important anatomical aspect found here was that the amount of radioactivity in the blood was negligible after the acupuncture points had been injected, thus suggesting that meridians and blood vessels are not related [4]. Chan and Bensoussan refer to components of the peripheral nervous system in relation to acupuncture points [5,6]. Similar relations have been presented by Bossy [7,8]. Melzack and Dung have considered acupuncture points to be related to trigger points [9] as well as to tenderness of the skin [10], respectively. In the past years Langevin and collaborators, based on post-mortem tissue sections of the arm, have described an association of acupuncture points to connective tissue structures [11]. 

The aim of this single case pilot study was to test the feasibility of a combined TCM and technological approach for the in-vivo characterization of acupuncture points using state-of-the art MRI imaging [12].

## Methods

Prior to in-vivo imaging a series of preliminary experiments were done in order to evaluate the interaction of the needles with the MRI scanner.

### Preliminary imaging sequences

**Figure 1:**
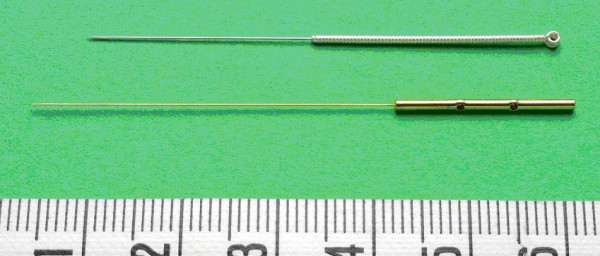
Comparison of the needles used in the preliminary testing. The upper needle is the usual stainless steel needle. The lower needle is the one made of gold. Both needle types made by Shenzhou, China. The size of the needles was: 25 × 0.25 mm for the stainless steel needle and 40 × 0.13 mm for the gold needle.

Preliminary experiments were done with conventional stainless steel acupuncture needles (Shenzhou, China, 0.25 × 25 mm). In the subsequent studies, acupuncture needles made of gold (Shenzhou, China, 0.13 × 40 mm) were used. The needles are shown in Figure 1. Both types of needles were obtained from Bacopa (Bacopa, Waidern 42, 4521 Schiedlberg, Austria [13]). The stability in place of the needles under operational MRI conditions was tested by placing them in a closed glass container. After evaluating the needle material, further preliminary experiments were done by inserting the needles into an onion and into a banana in order to evaluate the characteristics of the images being obtained.

#### In-vivo Acupuncture Imaging

Acupuncture procedures were carried out by a licensed medical practitioner (RM) on himself. The anthropometrical and functional characteristics of the subject are: age = 51 years, height = 174 cm, weight = 67 kg, BMI = 22.1, body fat = 12%, VO_2_max = 53 mL·kg^-1^·min^-1^. The study protocol was approved by the institutional ethics committee.

The acupuncture points chosen for the investigation were the Dai mai, i.e. gall bladder 26, and the Shen mai, i.e. bladder 62, points. A neutral needling technique was used. Neutral needling is known as Ping Bu Ping Xie and it refers to an equilibrated tonification and dispersion technique. The depth of needle insertion was done according to current descriptions [14]. The characteristics of the points can be found in [15]: "The Dai mai point is located in the depression one inch and eight fen below the region of the free ribs (11^th ^rib). This point corresponds to the intersection jiaohui point of the foot shao yang gall bladder channel and the girdling vessel. The Shen mai point is located in the depression five fen below the outer anklebone. It corresponds to the confluence-jiaohui point of the eight extraordinary vessels (yang motility vessel)". The term "fen" corresponds to 1/10 of a "cun" which is the traditional proportional body unit. One cun corresponds to the width of the interphalangeal joint of the thumb [14]. The imaging was done with only one needle at a time using the gold needles. The first imaging procedure was done on the Dai mai followed by the Shen mai point.

#### MRI imaging

MRI imaging was done on a Siemens Magnetom Avanto MR scanner using an array head and body coil. Mainly T1-weighted imaging sequences, as routinely used for patient exams, were used to obtain multi-slice images of the anatomy around the inserted needle. Imaging sequences were obtained using T1 weighted fast spin echo sequences in various orientations. The following imaging parameters were used: turbo factor: 3, TR: 500 or 721 ms, TE: 11 or 19 ms, flip angle of refocusing pulses: 150°, slice thickness: 3 or 2 mm, slice gap 10–30%, number of slices: 20–23, band width: 200 Hz/pixel, FOV: 400, 340 or 170 mm, acquisition matrix 320 or 384. Imaging was started within 5 minutes after needle insertion.

#### Image reconstruction

The abdominal MR slices corresponding to the Dai mai point were reconstructed using dedicated software from Nuclear Diagnostics (Hermes Medical Solutions AB, Skeppsbron 44, 111 30 Stockholm, SWEDEN; [16]. The procedure multimodality 3D was used to generate a volume rendered image.

## Results

### Preliminary imaging sequences

**Figure 2:**
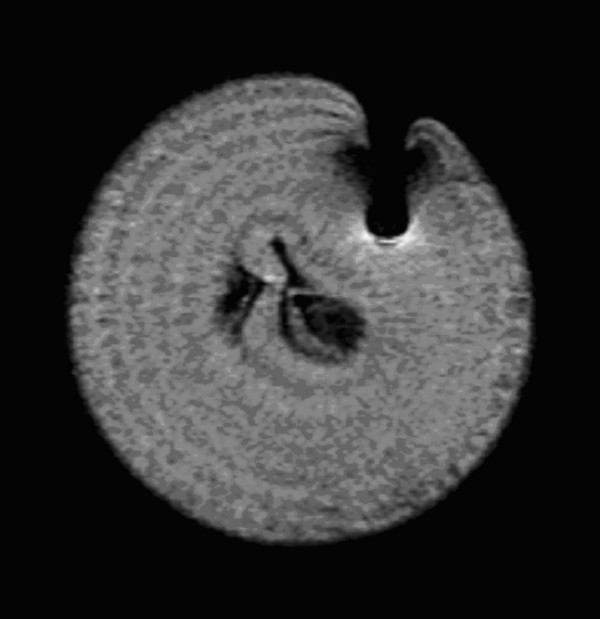
Preliminary imaging procedure using the gold needle inserted into an onion. Note the image attenuation area around the needle. In these experiments the length of the inserted portion corresponded to the length of the image attenuation effect. The width of the defect was much larger than the width of the gold needle.

**Figure 3:**
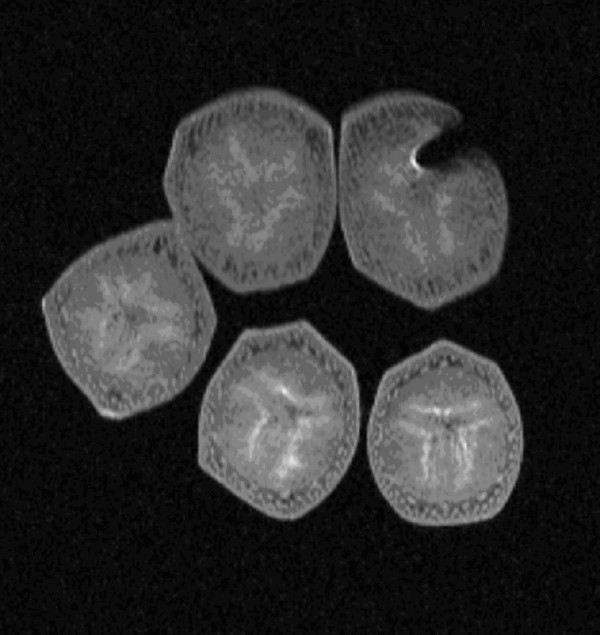
Preliminary imaging procedure using the gold needle inserted into a banana. Note the image attenuation area around the needle similar to that shown in figure 2. In these experiments the length of the inserted portion corresponded to the length of the image attenuation effect. The width of the defect was much larger than the width of the gold needle.

The preliminary experiments using the stainless steel needle showed erratic movements of the needle when the magnetic field was applied. Contrasting with this, the gold needle was stable in place in the closed glass container. For this reason all further experiments were done with the gold needles. In the experiments using an onion and a banana (Figures 2 and 3) an imaging artefact that produced an area of image attenuation around the needles was observed. In comparison to the diameter of the needles, the artefacts were about 6–8 times wider. On the other hand, the tip of the artefact area corresponded to the tip of the needle. The length of the artefact corresponded to the length of the inserted portion of the needle, thus making it possible to recognize which structures are found at this depth.

### In-vivo imaging of the Dai mai and Shen mai points

**Figure 4:**
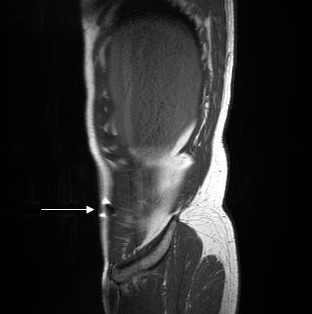
In-vivo localisation of the gold acupuncture needle inserted into the Dai mai point as seen in a saggital slice reconstruction. The white arrow simulates the direction of needle insertion. Image reconstruction was done with the J-Vision software of the TIANI workstation. MRI acquisition data: TR = 721, TE = 19, flip = 150, TH = 3, TF = 3, FOV = 370, MA = 320.

**Figure 5:**
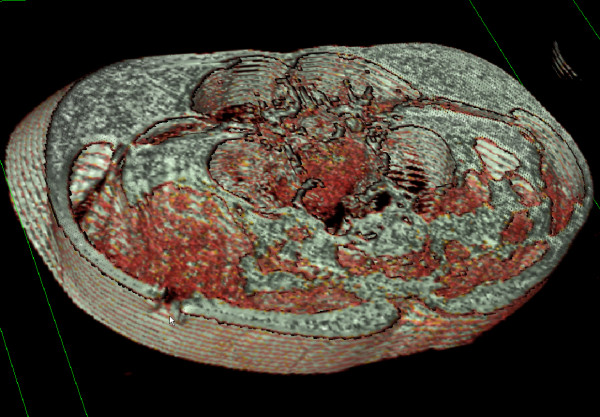
In-vivo localisation of the gold acupuncture needle inserted into the Dai mai point. The site of needle insertion is indicated by the white arrow. Image processing and reconstruction was done using the 3D volume rendering procedure of the multimodality module on the Hermes workstation. The color coding of the image allows the easy recognition of muscular structures of the abdomen. In this reconstruction the void effect of the gold needle is seen as a black area which reaches the muscular structures of the internal oblique muscle. Original MRI acquisition data: TR = 721, TE = 19, flip = 150, TH = 3, TF = 3, FOV = 370, MA = 320.

**Figure 6:**
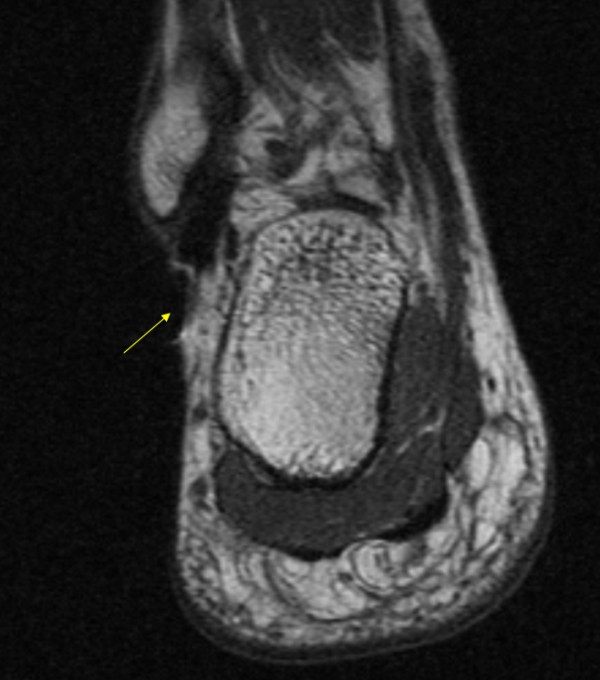
Localisation of the gold acupuncture needle inserted into the Shen mai point on the right ankle. A small image attenuation defect can be observed at the site where the needle was inserted (arrow). The tip of the needle was located close to the tendons of the peroneal muscles at the fibular malleolus. It should be noted, that the fibulo-maleolar ligament has not been reached. MRI acquisition data: TR = 500, TE = 11, flip = 150, TH = 2, TF = 3, FOV = 170, MA = 384.

**Figure 7:**
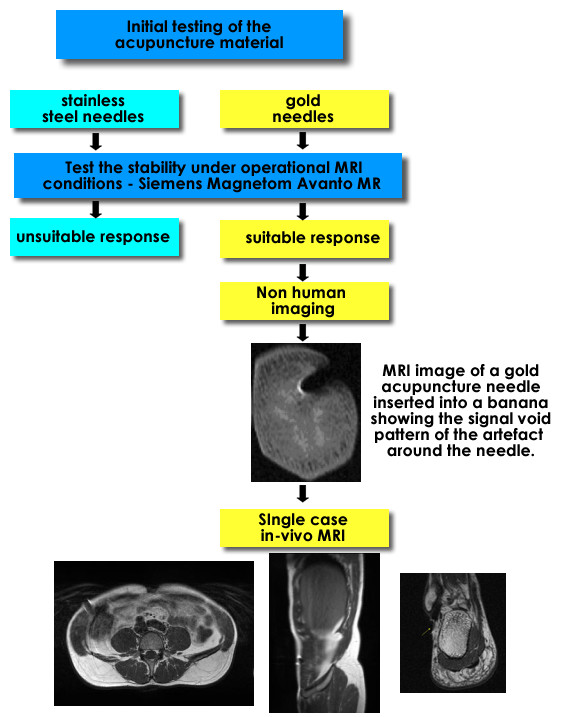
Flow chart showing a summary of the study including the preliminary evaluations, the non-human images, and the human in-vivo imaging.

During the study there were no side-effects such as localized hyperthermia, reddening of the skin or pain. A de qi sensation was felt at both points. The interaction of the needles with the MRI machine was limited to a slight bending of the handle of the needle. This bending did not compromise the stability of the needle. At the Dai mai point (lateral abdomen), the tip of the needle was in contact with the superficial fascia of the abdominal internal oblique muscle. Figures 4 and 5 show the position of the needle at the Dai mai point in 2 different reconstruction slices. Figure 4 shows a saggital slice and Figure 5, the 3D-volume rendered abdominal MRI image. At the Shen mai point (ankle), the artefact of the tip of the needle was located close to the tendons of the peroneal muscles at the fibular malleolus. The calcaneo-fibular ligament was untouched (Figure 6). The sequence of the experiments is summarized in Figure 7.

## Discussion

In this pilot investigation we have been able to show that it is feasible to indirectly identify the anatomical structures related to acupuncture points in-vivo using gold needles. Magnetizable steel acupuncture needles, e.g. stainless steel material, will be erratically displaced when tested under the MRI equipment and are therefore not suitable for MRI studies.

Metallic needles inserted into the body are known to produce artefacts in MRI imaging. This type of artefacts have been described as: "as a blooming ball-shaped signal void" and can be seen with biopsy needles [17]. In spite of having a central signal void characteristic, the position of the tip of the artefact corresponds to the tip of the needle and therefore to its length or depth of insertion. In our preliminary experiments we were able to confirm this by measuring the length of the inserted portion of the needle and the length of the artefact. For the evaluation of the in-vivo imaging of gold acupuncture needles one can rely on the tip of the void area as an adequate indicator of the underlying anatomy. In our study the tip of the inserted needles was in close relation to fasciae and tendons in the ankle, as well as to muscular structures in the abdomen. We are not aware of any similar studies that have used high resolution methods such as MRI for in-vivo imaging of acupuncture points in humans.

Due to the physical characteristics of the acupuncture needles, i.e. gold material and a small size, side effects related to heating during the MRI procedure were not found. Another interesting characteristic of gold needles is that they have a rougher surface as compared to stainless steel needles [11]. This might be the explanation for our finding of de qi sensation even though a neutral needling technique was used.

Previous investigations on the characterization of acupuncture points have used radionuclide methods which could clearly show that "meridian" flow was different to blood flow [4]. Other investigators have looked at acupuncture points in relation to components of the peripheral nervous system [5-8]. Melzack and Dung have considered acupuncture points to be related to trigger points [9] as well as to tenderness of the skin [10], respectively. In spite of these approaches, the anatomical basis of acupuncture points has not been defined. Langevin and associates have described an association of acupuncture points to connective tissue structures based on studies on post-mortem tissue sections of the arm [11]. Post-mortem studies, however, cannot obviate the problem of turgor loss as compared to in-vivo studies. Our study has shown that an in-vivo localization of anatomical structures related to acupuncture points on the abdomen and ankle is feasible using state-of-the-art MRI. An improved visualization of the results can be obtained by using 3D-volume rendering procedures.

In a recent publication Moncayo and Moncayo have described a general model of disease which is related to an eccentric body position [18]. This eccentric position affects both the feet and the head. Negative postural effects of this eccentric muscle position can be neutralized by using the acupuncture points described in this paper. It can thus be concluded, that the WOMED model of lateral tension is related to musculoskeletal structures and that at the same time, these structures, or acupuncture points as shown here, exert a regulating function on the system.

## Conclusion

An in-vivo investigation on the anatomical localisation and relationship of acupuncture points using MRI is feasible. The location of the tip of the needle corresponds to the tip of the signal void artefact seen on the images thus showing the underlying anatomy. These findings lend support to a musculoskeletal relation of the WOMED concept of lateral tension [18].

## Competing interests

The author(s) declare that they have no competing interests.

## Authors' contributions

RM planned the study, carried out the acupuncture procedures, wrote the draft of the manuscript. AR and CK were involved in the MRI procedures, data processing and image interpretation. MD carried out the 3D-image rendering on the Hermes workstation. All authors read and approved the final manuscript.

## Pre-publication history

The pre-publication history for this paper can be accessed here:
